# Dyspnea and Heart Failure: The Role of the Diaphragm

**DOI:** 10.2174/011573403X330739241216185852

**Published:** 2025-01-21

**Authors:** Pablo Marino Corrêa Nascimento, Mario Luiz Ribeiro, Bernardo Nascimento Lourenço, Humberto Villacorta, Antonio José Lagoeiro Jorge, Nazareth de Novaes Rocha, Wolney de Andrade Martins

**Affiliations:** 1 Postgraduate Program in Cardiovascular Sciences, Fluminense Federal University, Niterói, Rio de Janeiro, Brazil;; 2 National Institute of Cardiology, Rio de Janeiro, Rio de Janeiro State, Brazil

**Keywords:** Diaphragm, heart failure, dyspnea, exercise intolerance, ultrasound, inspiratory muscles, electronic databases

## Abstract

**Introduction:**

Dyspnea and exertional intolerance are the most common clinical manifestations of Heart Failure (HF). One of the possible mechanisms of both symptoms in HF patients is weakness of the inspiratory muscles.

**Aim:**

Because the diaphragm is the main inspiratory muscle, this review aimed to investigate the contribution of diaphragmatic function to the genesis of dyspnea or exercise intolerance in HF patients.

**Methods:**

Original articles, clinical trials, and cohort or case-control studies published between January 2003 and March 2023 were included. The population, variables, and outcome strategy were the basis of this review, including studies that assessed HF patients, diaphragmatic function, and dyspnea or exercise tolerance. The PubMed/MEDLINE, Embase, and BVS/LILACS databases were searched.

**Results and Discussion:**

A total of 353 articles were identified from electronic databases. After removing duplicate articles and screening based on titles, abstracts, and full texts, nine articles were included in the qualitative synthesis of this review. These studies were quite heterogeneous in their methodologies; however, most, except two, demonstrated an association among diaphragmatic dysfunction, dyspnea, and exertional intolerance in HF patients.

**Conclusion:**

Although few studies have assessed the contribution of diaphragmatic function to dyspnea and exertional intolerance in HF individuals, the vast majority of articles included in this review found such an association, especially when diaphragmatic function was assessed using ultrasound.

## 1 INTRODUCTION

Heart Failure (HF) is a global pandemic, affecting an estimated 64.3 million people worldwide. It has a prevalence of 1% to 3% of the adult population, an incidence of 1-20 cases per thousand, and incurs an annual cost of €25,500 per patient [1]. HF is a complex syndrome with high mortality [1, 2], comparable to several types of neoplastic diseases [3], and a considerably reduced quality of life [4].

Dyspnea, muscle fatigue, and exertional intolerance are the most common clinical manifestations of HF [5]. In addition to central changes, various abnormalities in skeletal muscles [6], particularly in the respiratory muscles [7], have been observed in HF. One of the possible mechanisms of dyspnea and reduced exertional tolerance in these individuals is weakness of the inspiratory muscles [8-10].

**Fig. (1):**
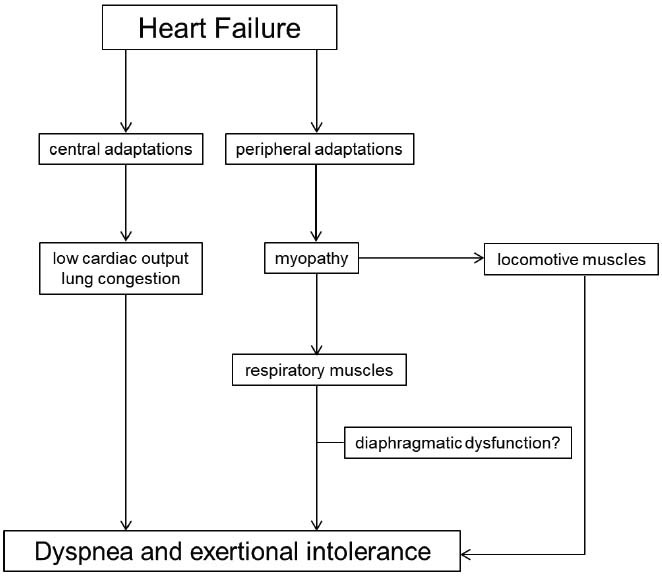
Pathophysiology of dyspnea and exertional intolerance in heart failure.

Considering that the diaphragm is the main inspiratory muscle [11, 12], this study aimed to investigate whether diaphragmatic dysfunction is present in HF and whether it contributes to dyspnea or exertional intolerance in these individuals. Such information may help in understanding the pathophysiology of this syndrome and in developing future therapeutic interventions (Fig. **1**).

## 2 METHODOLOGY

### 2.1 Eligibility Criteria

Original articles, clinical trials, and cohort or case-control studies published between January 2003 and March 2023 with full texts in English, Spanish, or Portuguese were included. The population, variables, and outcome strategy were the basis for this review. Considering this, we included studies that mentioned HF patients, assessed diaphragmatic function through invasive or noninvasive methods, and assessed dyspnea or exercise tolerance through functional tests or subjective scales.

Animal model studies, case reports, review articles, study protocols, and short communications, when published exclusively in abstract form, were excluded, as were duplicate articles.

### 2.2 Search Strategy

The search was conducted in the PubMed/MEDLINE and Embase and BVS/LILACS databases using the medical subject headings “dyspnea” or “exercise tolerance” and “heart failure” and “diaphragm.” The details of our search strategy are documented in the supplementary material.

### 2.3 Data Extraction

The articles were collected and analyzed by three independent researchers who initially selected them based on the title and abstract and then by reading the full material according to the eligibility criteria. In addition, a discussion was held among the researchers to resolve disagreements, with the subsequent inclusion of articles defined by consensus. After searching the references of those already accepted, we included further studies following the same criteria.

## 3 RESULTS

### 3.1 Identification and Selection of Studies

A total of 353 articles were identified from electronic databases. After removing duplicate articles and selecting them according to titles, abstracts, and full texts, nine articles were included in the qualitative synthesis of this review. The selection of included studies is detailed in the flowchart shown in Fig. (**2**).

### 3.2 General Characteristics of the Studies

Of the nine articles included, six were clinical studies [13-18] and three were mechanistic studies [19-21]. Eight observational studies (n=4 cross-sectional [18-21], n=3 retrospective [14-16], n=1 prospective [17]) and one randomized clinical trial [13] were included. Four studies included only HF patients with preserved Ejection Fraction (HFpEF) [14, 18-20], one study comprised HF patients with reduced Ejection Fraction (HFrEF) [14], and three studies involved HFrEF and HFpEF patients [15, 17, 21]. One study did not detail the HF phenotype of its participants [16]. Five studies used diaphragmatic ultrasound of the diaphragm as a method of assessing diaphragmatic function [14, 16, 18-21], three measured diaphragmatic force invasively using transdiaphragmatic pressure (Pdi) [19-21], and one employed surface Electromyography (EMG) on the diaphragm [17]. One study did not assess diaphragmatic function, but investigated pacemaker stimulation as a therapeutic strategy [13]. Regarding the assessment of dyspnea or exertion tolerance, five studies used the Six-minute Walk Test (6 MWT) [13-16, 21], three employed the Cardiopulmonary Exercise Test (CPET) [13, 18, 20], two included the New York Heart Association (NYHA) classification [13, 21], two involved the rating of perceived exertion [18, 19], and one included the Likert 5 score [17].

### 3.3 Clinical Studies

Beeler *et al.* evaluated 24 HFrEF outpatients selected for cardiac resynchronization therapy. These patients received implantation of an additional electrode in the left hemidiaphragm and were randomized into two groups: stimulation and control (no stimulation). At baseline and 9 weeks after the intervention, ventricular function, dyspnea according to the NYHA classification, and exercise tolerance were assessed using the distance covered in the 6 MWT and CPET on a lower limb cycle ergometer. Patients randomized to the pacing group showed improved dyspnea and left ventricular ejection fraction compared to the control group, as well as increased peak power and peak oxygen uptake (VO_2_ peak) in CPET. However, there was no difference between the groups in the distance covered in the 6 MWT [13].

Yamada *et al.* retrospectively evaluated 40 hospitalized HFpEF patients who underwent diaphragmatic ultrasound. The authors observed the prevalence of inspiratory muscle weakness, defined as a Maximum Inspiratory Pressure (MIP) below 70% of the predicted value in 27.5% of the sample. Patients with inspiratory muscle weakness had lower Vital Capacity (VC), lower limb muscle strength, lower handgrip strength, worse nutritional status, and shorter walking distance on the 6 MWT. MIP positively correlated with the distance walked in the 6 MWT, VC, lower limb muscle strength, and handgrip strength. The study observed a higher prevalence of inspiratory muscle weakness, restrictive pulmonary dysfunction, and a lower distance travelled in 6 MWT in patients with lower inspiratory diaphragm thickness (<3.9 mm) [14].

Miyagi *et al.* evaluated the diaphragmatic function using ultrasound in a retrospective study of 77 hospitalized HFpEF and HFrEF patients. The authors defined diaphragmatic dysfunction as an inspiratory thickness less than the median value of 4.0 mm and diaphragm atrophy as an expiratory thickness less than 2.0 mm. Thus, expiratory thickness corresponds to muscle mass, and inspiratory thickness corresponds to mass plus contractility, representing diaphragmatic function per se. The volunteers also underwent electrical bioimpedance analysis to quantify the skeletal muscle mass index. The authors compared patients with an inspiratory thickness of less than 4.0 mm with those with an inspiratory thickness equal to or greater than 4.0 mm. They observed diaphragmatic atrophy in 50% of patients and, more commonly, in patients with diaphragmatic dysfunction. Patients with diaphragmatic dysfunction, when compared to patients without dysfunction, had [a] lower distance travelled in the 6 MWT, [b] lower gait speed, [c] lower expiratory thickness, [d] lower inspiratory muscle strength assessed by MIP, [e] lower expiratory muscle strength assessed by Maximum Expiratory Pressure (MEP), [f] lower VC, [g] lower handgrip strength, [h] higher prevalence of respiratory muscle weakness, [i] higher prevalence of diaphragm atrophy, [j] higher prevalence of dynapenia, and [k] older age. Inspiratory diaphragm thickness was positively correlated with inspiratory and expiratory muscle strength, 6 MWT distance, gait speed, and handgrip strength. Hence, diaphragmatic dysfunction assessed using ultrasound represented the weakness of the inspiratory musculature and predicted exertional intolerance regardless of the presence of pulmonary dysfunction, dynapenia, or sarcopenia in HF [15].

In a retrospective study of 62 patients hospitalized with HF, Kinugasa *et al.* used ultrasound of the diaphragm and similarly defined diaphragmatic dysfunction as an inspiratory thickness of less than a median value of 4.0 mm. The patients were divided into two groups: [a] dynapenia including those with reduced muscle strength, but no reduction in muscle mass, and [b] sarcopenia comprising those with a reduction in strength and muscle mass, according to the Asian Work Group for Sarcopenia criteria [22]. The prevalences of sarcopenia and dynapenia were 32.3% and 40.3%, respectively. Dynapenia or sarcopenia patients were older, had lower knee extensor muscle strength, had worse diaphragmatic function, and walked shorter distances in the 6 MWT than those without dynapenia or sarcopenia. The reduced diaphragmatic function was associated with an additional decrease in the distance walked in the 6 MWT in both dynapenia and sarcopenia patients. In multivariate analysis, diaphragmatic dysfunction, older age, and lower knee extensor muscle strength were independent determinants of reduced 6 MWT distance in HF patients with dynapenia or sarcopenia [16].

In a prospective study of 25 acute HF patients (45% with HFpEF), Luiso *et al.* performed surface EMG on the diaphragm, scalene, and pectoralis minor, and calculated the dyspnea score (Likert 5 scale). Participants were assessed at three time-points: on the day of admission and the second and fifth days of hospitalization. The dyspnea score decreased progressively over the three measurements, and there was a positive correlation between the diaphragm and scalene EMG activity index and dyspnea score; thus, the higher the diaphragm and scalene activity, the greater the severity of dyspnea assessed by the score. In contrast, pectoralis minor EMG did not correlate with dyspnea score [17].

Andriopoulou *et al.* compared 25 HFpEF outpatients and 25 healthy controls in a cross-sectional study using ultrasound of the quadriceps and the diaphragm [assessed for motion (excursion)]. The volunteers underwent CPET. Compared to the control group, HFpEF patients had a lower diaphragm excursion, quadriceps thickness, and rectus femoris cross-sectional area. In the CPET, a negative correlation was observed between the VO_2_ peak and the classification of perceived dyspnea. In contrast, there was a positive correlation between VO_2_ peak and rectus femoris cross-sectional area, quadriceps thickness, and diaphragmatic excursion. In addition, greater diaphragm excursion was associated with a greater rectus femoris cross-sectional area and greater quadriceps thickness. Multiple linear regression showed that diaphragm excursion was the main parameter associated with VO_2_ peak in the total and HFpEF groups; in the control group, the main parameters were diaphragm excursion and quadriceps thickness (Table **1**) [18].

Table **1** summarizes the main characteristics and most relevant results of the clinical studies included in this review.

### 3.4 Mechanistic Studies

Nava *et al.* compared 11 hospitalized HFrEF patients and 10 healthy controls [19]. Dyspnea was assessed by rating perceived exertion using the Borg scale [20-23] and diaphragmatic strength by measuring Pdi. They used the diaphragmatic Pressure-time Product per minute (PTPdi/min) as an index of metabolic and exertional consumption of the diaphragm. They also assessed the effect of changing from the sitting to the supine position and then applying Non-invasive Ventilation (NIV) in the supine position. A lower Pdi was found in the HF patients than in the control group, which decreased further with a change in the supine position. The PTPdi/min in the sitting position was higher in HFrEF patients than in the control group, and switching to the supine position further increased the PTPdi/min in this group. Switching to the supine position in HFrEF patients increased dyspnea and pulmonary resistance and decreased lung compliance. There was a positive correlation between the increase in dyspnea in the supine position and the percentage increase in PTPdi/min in HFrEF patients. NIV decreased the PTPdi/min in HFrEF patients, and there was a positive correlation between the reduction in PTPdi/min and the reduction in dyspnea. In the control group, switching to the supine position did not affect any variables. Hence, an increased diaphragmatic effort was strongly correlated with orthopnea in the HFrEF group [19].

Dayer *et al.* studied 12 HFrEF outpatients and 12 healthy controls who underwent an incremental exercise test on a lower limb cycle ergometer until exhaustion. The transdiaphragmatic pressure of the volunteers was measured after bilateral anterolateral phrenic nerve magnetic stimulation (twitch Pdi) at rest and after maximal exercise (25 and 35 min). At rest, no differences were observed in the volitional (MIP) and non-volitional tests of respiratory muscle strength (esophageal pressure during maximal sniffing). Similarly, no decrease in twitch Pdi after exercise was noted in HFrEF patients or the control group. Therefore, low-frequency diaphragmatic fatigue was not observed after maximal incremental cycle ergometer exercises [20].

Spiesshoefer *et al.* compared 30 HF outpatients, 22 HFrEF outpatients, 8 HFpEF outpatients, and 19 healthy controls. The volunteers underwent MIP and MEP measurements, diaphragmatic ultrasound, and magnetic stimulation of the posterior cervical phrenic nerve. Compared to the control group, HFrEF and HFpEF patients had lower MIP, MEP, Forced Vital Capacity (FVC), and diaphragm thickness (40%). Diaphragm strength characterized by Pdi in response to supramaximal phrenic nerve magnetic stimulation was 35% lower in HFrEF patients and 55% lower in HFpEF patients compared to the control group. There were no differences in diaphragmatic ultrasound parameters between the HFrEF and HFpEF groups. In HFrEF patients, NYHA functional class was negatively correlated with MIP, MEP, and FVC, and the distance walked in the 6 MWT was positively correlated with MIP and FVC. In addition, HFrEF patients had fewer diaphragm excursions (40%) than the control group. There was no correlation between diaphragmatic ultrasound and NYHA functional class or the N-terminal fraction of the B-type Natriuretic Peptide (NTproBNP). No correlation was found between invasively measured respiratory muscle strength and 6 MWT distance, NYHA functional class, or NTproBNP (Table **2**) [21].

Table **2** summarizes the main characteristics and the most relevant results of the mechanistic studies included in this review.

## 4 DISCUSSION

Dyspnea, muscle fatigue, and exertional intolerance are the most common clinical manifestations of HF [5]. Inspiratory muscle weakness is one of the possible mechanisms responsible for dyspnea [8, 9] and reduced exercise tolerance [10, 15]. HF patients with reduced inspiratory muscle strength have lower VO_2_ peak values [24, 25], worse functional classes according to the NYHA classification [26, 27], and increased mortality [7]. Conversely, inspiratory muscle training in HF individuals has resulted in numerous benefits, such as increased MIP [28-36], increased exercise tolerance assessed by the distance travelled in the 6 MWT [28, 29, 31, 33] and direct measurement of VO_2_ peak [28-30, 32, 33], improvement of dyspnea [28-31], improvement of quality of life [28, 32, 33, 36], and improvement of ventilatory efficiency [32, 33, 35].

The diaphragm is the main inspiratory muscle [11, 12], and diaphragm biopsy studies have revealed a variety of histological abnormalities in HF patients [37]. In an animal model, atrophy of type I muscle fibers was observed [38], potentially indicating a part of the generalized muscle disorder previously described in HF [6].

The studies included in this review were few and heterogeneous in their methodology. However, most studies have demonstrated an association among diaphragmatic dysfunction, dyspnea, and exertional intolerance. In HFpEF patients, diaphragmatic dysfunction in the ultrasound was associated with lower inspiratory muscle strength and shorter distance travelled in the 6 MWT [14]. Similarly, in HFpEF and HFrEF patients, diaphragmatic dysfunction in the ultrasound was associated with a shorter distance travelled in the 6 MWT [15, 16], lower inspiratory muscle strength [15], and a higher prevalence of diaphragmatic atrophy [15]. In these patients, a positive correlation between inspiratory thickness and the distance covered in the 6 MWT and inspiratory muscle strength was also observed [15]. The main determinants of the lower distance covered in the 6 MWT were older age, lower limb muscle strength, and lower inspiratory diaphragm thickness [16].

Although the 6 MWT has clinical and prognostic importance [39], the CPET is the gold standard for assessing exercise tolerance and maximal aerobic power, particularly in HF patients. In the only study that assessed the impact of diaphragmatic function on CPET, a positive correlation was observed between diaphragmatic excursion and VO_2_ peak [18], corroborating the impact of diaphragmatic function assessed using ultrasound on exercise tolerance.

Nava *et al.* invasively assessed diaphragmatic function and found lower strength and more significant diaphragmatic effort in HFrEF patients in the sitting position. This diaphragmatic effort was accentuated in the supine position and reduced with NIV. A correlation was also found between increased dyspnea and diaphragmatic effort in the supine position, suggesting that diaphragmatic effort is related to orthopnea, a common symptom in HF patients [19].

In patients hospitalized with acute HF, a correlation between diaphragmatic activity on EMG and dyspnea score was noted, which decreased over the days of hospitalization owing to therapeutic measures and clinical stabilization [17].

Finally, electrical stimulation of the diaphragm with an implanted pacemaker at the time of cardiac resynchronization resulted in an increased VO_2_ peak and maximal power in the CPET and reduced dyspnea in HFrEF patients [13].

In contrast, two of the nine studies found no association between diaphragmatic function and the outcomes investigated in this review. Dayer *et al.* documented no reduction in respiratory muscle strength or low-frequency diaphragmatic fatigue in HFrEF patients after maximal CPET using a lower limb cycle ergometer. However, it was not established whether there was a correlation between respiratory muscle strength and VO_2_ peak [20]. Spiesshoefer *et al.* found no correlation between sonographic parameters of diaphragmatic function and NYHA classification. They did not find a correlation between the invasive measurement of inspiratory muscle strength and NYHA classification or distance walked during the 6 MWT. Nonetheless, the authors did not describe whether there was a correlation between sonographic parameters and distance travelled in the 6 MWT [21].

## CONCLUSION

Although few studies have assessed the contribution of diaphragmatic function to dyspnea and exertional intolerance in HF patients, the vast majority of articles included in this review found such an association, especially when diaphragmatic function was assessed using ultrasound. Additional studies are needed to further explore this hypothesis, preferably by correlating diaphragmatic function with more objective CPET parameters, which is the gold standard method for assessing exercise tolerance and maximal aerobic power.

## Figures and Tables

**Fig. (2):**
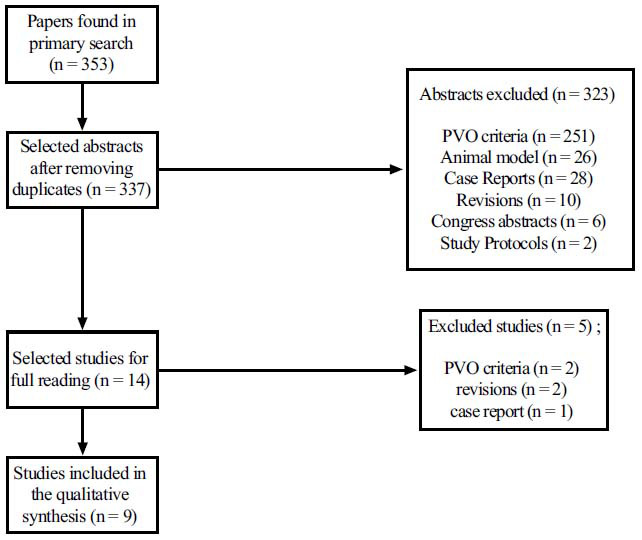
Flowchart of study selection. **Abbreviation:** PVO: population, variable of interest, outcome.

**Table 1:** Characteristics and results of the included clinical studies.

**Clinical Studies**	**Population**	**Assessment of Diaphragmatic Function**	**Intervention on the Diaphragm**	**Assessment of Dyspnea or Exertional Intolerance**	**Results**
Beeler *et al.* [13]	HFrEF (n = 24)	-	Pacemaker stimulation	NYHA CPET 6 MWT	Decreased NYHA; Increased VO_2_ peak; Did not modify distance in the 6 MWT
Yamada *et al.* [14]	HFpEF (n = 40)	US	-	6 MWT	Lower inspiratory thickness associated with shorter distance in 6 MWT
Miyagi *et al.* [15]	HFrEF and HFpEF (n = 77)	US	-	6 MWT	Correlation between inspiratory thickness and distance in the 6 MWT
Kinugasa *et al.* [16]	HF (n = 62)	US	-	6 MWT	Inspiratory thickness was an independent predictor of shorter 6MWT distance in individuals with dynapenia or sarcopenia.
Luiso *et al.* [17]	Acute HF (n = 25)	EMG	-	Likert 5	Correlation between EMG and dyspnea score
Andriopoulou *et al.* [18]	HFpEF (n = 25) CG (n = 25)	US	-	CPET	Correlation between diaphragm excursion and VO_2_ peak

**Abbreviations:** EMG, electromyography; HF, heart failure; HFpEF, heart failure with preserved ejection fraction; HFrEF, heart failure with reduced ejection fraction; CG, control group; NYHA, New York heart association; US, ultrasonography; CPET, cardiopulmonary exercise test; 6 MWT: six-minute walk test; VO_2_ peak: peak oxygen consumption on exertion.

**Table 2:** Characteristics and results of the included mechanistic studies.

**Studies**	**Population**	**Assessment of Diaphragmatic Function**	**Intervention on the Diaphragm**	**Assessment of Dyspnea or Exertional Intolerance**	**Results**
Nava *et al.* [19]	HFrEF (n = 11) CG (n = 10)	PTPdi/min	-	Rating of perceived exertion	Correlation between increased dyspnea in the supine position and increased PTPdi/min in HFrEF patients. Correlation between PTPdi/min reduction and dyspnea reduction with NIV in the supine position.
Dayer *et al.* [20]	HFrEF (n = 12) CG (n = 12)	twitch Pdi	-	CPET	Did not document diaphragmatic fatigue after maximal exercise.
Spiesshoefer *et al.* [21]	HFrEF (n = 22) HFpEF (n = 8) CG (n = 19)	US twitch Pdi	-	NYHA 6 MWT	No correlation between US and NYHA. No correlation between twitch Pdi and distance in 6MWT. No correlation between twitch Pdi and NYHA.

**Abbreviations:** HFpEF, heart failure with preserved ejection fraction; HFrEF, heart failure with reduced ejection fraction; CG, control group; NYHA, New York Heart Association; PTPdi/min, diaphragmatic pressure-time product per minute; US, ultrasonography; NIV, noninvasive ventilation; CPET, cardiopulmonary exercise test; 6 MWT: six-minute walk test; twitch Pdi, transdiaphragmatic pressure after phrenic nerve magnetic stimulation.

## LIST OF ABBREVIATIONS

CPET: Cardiopulmonary Exercise Test
EMG: Electromyography
FVC: Forced Vital Capacity
HF: Heart Failure
MEP: Maximum Expiratory Pressure
MIP: Maximum Inspiratory Pressure
NIV: Non-invasive Ventilation
VC: Vital Capacity
