# Supplementary material for: Dyspnea and Heart Failure: The Role of the Diaphragm
Source: Curr Cardiol Rev. 2025 Jan 21;21(3):E1573403X330739. doi: 10.2174/011573403X330739241216185852 (PMC12172216; doi:10.2174/011573403X330739241216185852)
Supplement: Supplementary file 1 — Supplementary material is available on the publisher’s website along with the published article. [file CCR-21-3-E1573403X330739_SD1.pdf]

Supplementary Material

Dyspnea and Heart Failure: The Role of the Diaphragm

Pablo Marino Corrêa Nascimento[1,2,*], Mario Luiz Ribeiro[1], Bernardo Nascimento Lourenço[1], Humberto Villacorta[1], Antonio José Lagoeiro Jorge[1], Nazareth de Novaes Rocha[1], Wolney de Andrade Martins[1]

[1]*Department of Cardiovascular Sciences, Fluminense Federal University, Niterói, Rio de Janeiro, Brazil;* [2]*National Institute of Cardiology, Rio de Janeiro, Rio de Janeiro State, Brazil*

PUBMED: (((("Dyspnea"[MeSH Terms] OR "breath shortness"[Title/Abstract] OR "Breathlessness"[Title/Abstract] OR "Dyspneas"[Title/Abstract] OR "shortness of breath"[Title/Abstract] OR (("Breath"[All Fields] OR "breathe"[All Fields] OR "breathed"[All Fields] OR "breathes"[All Fields] OR "breathings"[All Fields] OR "breaths"[All Fields] OR "respiration"[MeSH Terms] OR "respiration"[All Fields] OR "breathing"[All Fields]) AND "rest dyspnea"[Title/Abstract]) OR "dyspnea rest"[Title/Abstract] OR (("Dyspneas"[All Fields] OR "dyspnoea"[All Fields] OR "Dyspnea"[MeSH Terms] OR "Dyspnea"[All Fields]) AND "Rest"[Title/Abstract]) OR ("exercise tolerance"[MeSH Terms] OR "tolerance exercise"[Title/Abstract])) AND ("heart failure"[MeSH Terms] OR "cardiac failure"[Title/Abstract] OR "congestive heart failure"[Title/Abstract] OR "heart failure congestive"[Title/Abstract] OR "heart failure left sided"[Title/Abstract] OR "heart failure left sided"[Title/Abstract] OR "left sided heart failure"[Title/Abstract] OR "left sided heart failure"[Title/Abstract] OR "myocardial failure"[Title/Abstract])) OR ("heart failure, systolic"[MeSH Terms] OR (("heart failure"[MeSH Terms] OR ("Heart"[All Fields] AND "Failure"[All Fields]) OR "heart failure"[All Fields] OR ("Heart"[All Fields] AND "Failures"[All Fields]) OR "heart failures"[All Fields]) AND "Systolic"[Title/Abstract]) OR (("systole"[MeSH Terms] OR "systole"[All Fields] OR "systoles"[All Fields] OR "Systolic"[All Fields] OR "systolically"[All Fields]) AND "heart failures"[Title/Abstract]) OR "systolic heart failure"[Title/Abstract] OR "heart failure reduced ejection fraction"[Title/Abstract]) OR ("heart failure, diastolic"[MeSH Terms] OR "diastolic heart failures"[Title/Abstract] OR "heart failure preserved ejection fraction"[Title/Abstract] OR "heart failure normal ejection fraction"[Title/Abstract] OR "diastolic heart failure"[Title/Abstract])) AND ("Diaphragm"[MeSH Terms] OR "contraceptive devices, female"[MeSH Terms] OR "diaphragm respiratory"[Title/Abstract] OR (("eur med j respir"[Journal] OR "Respiratory"[All Fields]) AND "Diaphragms"[Title/Abstract]) OR "respiratory diaphragm"[Title/Abstract] OR "Diaphragms"[Title/Abstract])) AND (2003:2023[pdat])

EMBASE: ('heart failure'/exp OR 'backward failure, heart' OR 'cardiac backward failure' OR 'cardiac failure' OR 'cardiac incompetence' OR 'cardiac insufficiency' OR 'cardial insufficiency' OR 'chronic heart failure' OR 'chronic heart insufficiency' OR 'heart backward failure' OR 'heart failure' OR 'heart incompetence' OR 'heart insufficiency' OR 'insufficientia cardis' OR 'myocardial failure' OR 'myocardial insufficiency' OR 'systolic heart failure'/exp OR 'heart failure, systolic' OR 'systolic heart failure' OR 'diastolic heart failure'/exp OR 'diastolic heart failure' OR 'heart failure, diastolic' OR 'congestive heart failure'/exp OR 'cardiac congestive failure' OR 'congestive cardiac failure' OR 'congestive heart failure' OR 'congestive heart insufficiency' OR 'heart failure, congestive' OR 'heart failure with preserved ejection fraction'/exp OR 'heart failure with preserved ejection fraction' OR 'heart failure with reduced ejection fraction'/exp OR 'heart failure with reduced ejection fraction') AND ('diaphragm'/exp OR 'diaphragm' OR 'diaphragm muscle' OR 'diaphragma' OR 'diaphragmatic muscle') AND ('dyspnea'/exp OR 'breathing difficulties' OR 'breathing difficulty' OR 'breathlessness' OR 'difficult breathing' OR 'difficult respiration' OR 'difficulty breathing' OR 'dyspnea' OR 'dyspneas' OR 'dyspneic syndrome' OR 'dyspnoea' OR 'dyspnoeae' OR 'dyspnoeas' OR 'labored respiration' OR 'laboured respiration' OR 'shortness of breath' OR 'exercise tolerance'/exp OR 'exercise tolerance' OR 'tolerance, exercise')

#1 AND (2003:py OR 2004:py OR 2005:py OR 2006:py OR 2007:py OR 2008:py OR 2009:py OR 2010:py OR 2011:py OR 2012:py OR 2013:py OR 2014:py OR 2015:py OR 2016:py OR 2017:py OR 2018:py OR 2019:py OR 2020:py OR 2021:py OR 2022:py OR 2023:py)

BVS/LILACS: (dispneia OR tolerância ao exercício) AND (insuficiência cardíaca) AND (diafragma) AND (year_cluster:[2003 TO 2023])

1875-6557 /25 ©2025 Bentham Science Publishers
